# Longitudinal description of health-related quality of life and depressive symptoms in polyQ spinocerebellar ataxia patients

**DOI:** 10.1007/s00415-025-13024-0

**Published:** 2025-04-09

**Authors:** Audrey Iskandar, Maresa Buchholz, Iris Blotenberg, Tanja Schmitz-Hübsch, Jennifer Faber, Heike Jacobi, Feng Xie, Marcus Grobe-Einsler, Thomas Klockgether, Bernhard Michalowsky, ESMI and EUROSCA Study groups, Kathrin Reetz, Mafalda Raponso, Carlos Gonzales, Berkan Koyak, Demet Oender, Luís Pereira de Almeida, Patrick Silva, Joana Afonso Ribeiro, Andreas Thieme, Friedrich Erdlenbruch, Jeroen de Vries, Chiadikaobi Onyike, Paola Giunti, Hector Garcia-Moreno, Gülin Öz, Almut Turid Bischoff, Bart van de Warrenburg, Judith van Gaalen, Jon Infante, Leire Manrique, Ludger Schöls, Olaf Riess, Sophie Tezenas du Montcel, Peter Bauer, Paola Giunti, Arron Cook, Robyn Labrum, Michael H. Parkinson, Alexandra Durr, Alexis Brice, Perrine Charles, Cecilia Marelli, Caterina Mariotti, Lorenzo Nanetti, Marta Panzeri, Maria Rakowicz, Anna Sulek, Anna Sobanska, Holger Hengel, Laszlo Baliko, Bela Melegh, Alessandro Filla, Antonella Antenora, José Berciano, Dagmar Timmann, Sandra Szymanski, Sylvia Boesch, Jun-Suk Kang, Massimo Padolfo, Jörg B. Schulz, Sonia Molho, Alhassane Diallo

**Affiliations:** 1 German Center for Neurodegenerative Diseases (DZNE), Site Rostock/Greifswald, Patient-Reported Outcomes & Health Economics Group, Ellernholzstrasse 1-2, 17489 Greifswald, Germany; 2 German Center for Neurodegenerative Diseases E.V. (DZNE), Site Bonn, Bonn, Germany; 3 Department of Neurology, University of Bonn, Bonn, Germany; 4 Experimental and Clinical Research Center, A cooperation of Max-Delbrueck Center of Molecular Medicine and Charité, Universitätsmedizin Berlin, Berlin, Germany; 5 Department of Neurology, University Hospital Heidelberg, Heidelberg, Germany; 6 Centre for Health Economics and Policy Analysis, Department Health Research Methods, Evidence and Impact, McMaster University, Hamilton, Canada

**Keywords:** Health-related quality of life, Psychologic well-being, Ataxia, Rare disease, Genetic disorder, Patient-centered care

## Abstract

**Introduction:**

Due to limited treatment options, managing symptoms has dominated care for Spinocerebellar Ataxia (SCA). Little attention has been given to health-related quality of life (HRQoL) and depressive symptoms experienced by patients across disease duration.

**Objective:**

To investigate the course of HRQoL and the severity of depressive symptoms in SCA from disease onset to 26 years after onset and identify influencing factors.

**Methods:**

We analyzed data from two longitudinal SCA cohorts, the EUROSCA (European Spinocerebellar Ataxia Registry) and ESMI study (European Spinocerebellar Ataxia Type 3/Machado-Joseph Disease Initiative). Multilevel mixed-effects models were employed to demonstrate the course of HRQoL and depressive symptoms severity to investigate the role of disease progression with disease duration as a predictor of interest, along with time-varying clinical variables and time-fixed covariates.

**Results:**

Seven hundred seventy four participants (M_age_ = 50.8 ± 13.4; 48.6% female) were included. HRQoL consistently decreased throughout disease duration across all SCA subtypes, but the decline was smallest in SCA6. The decrease in HRQoL was explained by ataxia and depression severity and driven by increasing problems with self-care, usual activities and mobility. Depressive symptoms significantly increased in SCA2 and 3 only, with a trend toward slight improvement in SCA6.

**Conclusions:**

The trend direction of HRQoL and its significant association with the severity of ataxia symptoms align with the literature. The rapid worsening of self-care problems, the differential associations between depression and HRQoL sub-dimensions in different SCA subtypes, and the unexplainable resilience may warrant a deeper look at patient-specific intra- and interpersonal factors.

**Supplementary Information:**

The online version contains supplementary material available at 10.1007/s00415-025-13024-0.

## Introduction

Spinocerebellar Ataxia (SCA) is a group of inherited genetic diseases primarily affecting the cerebellum, with more than 50 different types. [1] The most common and studied subtypes are SCA1, SCA2, SCA3 (also known as Machado-Joseph Disease) and SCA6, with a prevalence of 1–5:100,000 [2, 3]. This subgroup of SCA shares a common disease mechanism, in which CAG repeats within specific genes leads to the production of abnormal proteins (polyglutamine or polyQ SCA) [4, 5]. Difficulties experienced by SCA patients generally involve coordination, resulting in gait disturbances and impaired fine motor skills, as well as accompanying symptoms such as dysphagia (swallowing difficulties), dysarthria (speech difficulties), and oculomotor impairments. The age of onset varies between subtypes but typically starts to manifest in adulthood [6]. Early onset is associated with more CAG repeats in the expanded allele. [7–9]. The progression and symptoms present differently in each SCA subtype. Among the four investigated SCA subtypes, SCA 6 is known to manifest the latest and progress the slowest with a pure cerebellar phenotype [10]. Diallo et al. also revealed that SCA 6 has the highest and SCA1 has the lowest life expectancy [11].

Without curative treatments, the current focus lies on symptom management and improving health-related quality of life (HRQoL) [12]. For example, Weber et al. [13] identified that mental health and weight contributed to more intense HRQoL decline in SCA, especially in male patients with early disease onset. Jacobi et al. [14] showed that depressive symptoms assessed with Patient Health Questionnaire-9 (PHQ-9) increase with the presence of cognitive impairment. Other studies revealed that HRQoL differs across SCA subtypes [15] and is associated with physical function [16] and the presence of a carer [17].

Widening the focus on depressive symptoms in SCA is equally important, as approximately one-third of individuals with SCA experience clinically relevant depression [18, 19], increasing impairments of Activities in Daily Livings (ADLs), dependency, caregiver burden [20] and, thereby possibly, HRQoL. Lin et al. in 2018 [21] demonstrated a bidirectional relationship between and ataxia severity. Other factors, such as age of onset and diagnosis acceptance, might also play a significant role in HRQoL and depressive symptoms but have not been explored. People in different life stages have different goals and priorities. The inability to pursue personal goals would result in mental health deterioration [22]. Thus, younger patients with early onset might face tremendous challenges related to their developmental tasks [18], such as adjusting to a potentially shortened life span (early), loss of independence and opportunities. All of this occurred while emotional regulation skills were still under development. Moreover, investigating the trajectory of depressive symptoms’ severity is even more crucial in maintaining overall quality of life, as the progressive cerebellar degeneration disrupts cerebello-limbic circuits involved in mood regulation potentially leading to depression [18, 23]. Thereby, early intervention could potentially improve patients’ outcome and ease overall disease burden.

While the overall rate of annual decline in physical symptoms is well described in SCAs [10, 11, 24], a deeper understanding of the evolution of HRQoL and its association with depressive symptoms is crucial to inform clinical practice and guide future research. Therefore, this study aimed to assess the change in HRQoL and depressive symptoms over time from disease onset in a large multicenter cohort of SCA1, SCA2, SCA3 and SCA6 patients.

## Methods

### Study design and sample

This study used retrospective data from two large longitudinal SCA studies: the European Spinocerebellar Ataxia Registry (EUROSCA,* N* = 524) [24], containing data from patients with SCA 1, 2, 3, and 6, and the European Spinocerebellar Ataxia Type 3/Machado-Joseph Disease Initiative (ESMI, *N* = 250) with data from patients with SCA 3. Participants of ESMI were included at 11 European and an additional three US study sites since 2016 [25]. EUROSCA was performed at 17 European sites [26, 27] from 2005 until 2016 [26]. We used data from the baseline (*n* = 774) and three consecutive annual follow-up assessments (*n* = 652 1, *n* = 533 2, and *n* = 424 3 years after baseline) of both studies.

ESMI and EUROSCA were approved by the ethics committees of the participating centers and have been performed following the ethical standards of the Declaration of Helsinki. Written informed consent was obtained from all study participants. EUROSCA was registered with a ClinicalTrials.gov number (NCT02440763).

### Data assessment

Participants completed a standardized battery of health-related questionnaires (i.e., EQ-5D-3L, PHQ-9), sociodemographic variables (i.e., age, sex, onset age) and clinical tests via face-to-face consultations carried out by health professionals at baseline and every annual follow-up study center visit.

The EQ-5D-3L, a widely validated measure, was used to assess HRQoL [28]. The EQ-5D-3L includes a descriptive system with five HRQoL dimensions (mobility, self-care, usual activities, pain/discomfort, and anxiety/discomfort) with three severity levels (no, some, extreme problems). The responses from each dimension can be converted into a single health utility index ranging from 0 (death) to 1 (full health) using the European value set [29].

The PHQ-9 questionnaire, used in this study, is a nine-item screening measure to assess depressive symptom severity [30], ranging from 0 to 27, with higher scores indicating more depressive symptoms. The score can be categorized into the following four groups: 0–4, none to minimal depression, 5–9 mild depression, 10–14 moderate depression, and ≥ 15 severe depression [31], with scores higher than 9 indicating clinically significant diagnosis when used in screening.

The Scale for the Assessment and Rating of Ataxia (SARA) was designed to assess ataxia severity. SARA includes eight items (gait, stance, sitting, speech, finger-chase test, nose-finger test, fast alternating movements, and heel-shin test) and ranges from 0 (no ataxia) to 40 (most severe ataxia). We categorized the scores according to Traschütz et al. 2023: mild < 10, moderate 10–25, and advanced > 25 [32]. The frequency distribution of answer behavior can be found in Supplementary Tables 3 and 4.

### Data preparation: disease duration & age of onset

Participants without information on actual age, onset age, or both were excluded. We calculated disease duration by subtracting the age of onset from the age at data collection, ranging from 0 to 26 years. (see Supplementary Table 1 for distribution of onset). The reported onset of gait disturbances is defined as the age of onset. To investigate the impact of age of onset on HRQoL and depression, we split the age of onset into early, mid, and late-onset. Considering the high heterogeneity, the age of onset was divided into tertiles for each SCA subtype in three different variables (see Table 1). EQ-5D-3L domain responses of 2 (some problems) and 3 (severe problems) were merged to provide a more straightforward interpretation of problem probability at different disease phases. However, separate domain-level trajectories were included in the supplementary material.

### Statistical analyses

The cross-sectional descriptive statistics were computed to characterize the participants at baseline. Due to the skewed distribution, we used the Kruskal–Wallis test and the Chi^2^ test of independence to compare the baseline descriptive statistics between SCA subtypes.

To investigate the role of disease duration on HRQoL and depression, we calculated linear and ordinal logistic mixed-effect models with a random intercept for each individual [33] over the period of four annual timepoints. The additional variables chosen as predictors (including time-varying, i.e., changes over time and time-fixed, and i.e., stays constant, covariates) are factors in disease duration that might affect the trajectory of HRQoL differently. To visualize how the probability of EQ-5D subdimension rating changed with disease progression, we conducted a similar mixed-effect analysis for each EQ-5D subdimension to show changes in probabilities of each level being chosen over time. An additional model with the depression score (PHQ-sum score) as the outcome was generated. EQ-5D subdimensions were added to the model to investigate its association with depressive symptom severity and address the potential bidirectional relationship. The same model specifications were otherwise used as in the full model. Microsoft Excel (Version 2016) and STATA 18.0 (StataCorp, 2023) were used in the data exploration and analysis, respectively.

## Results

### Sample characteristics at baseline

**Fig. 1:**
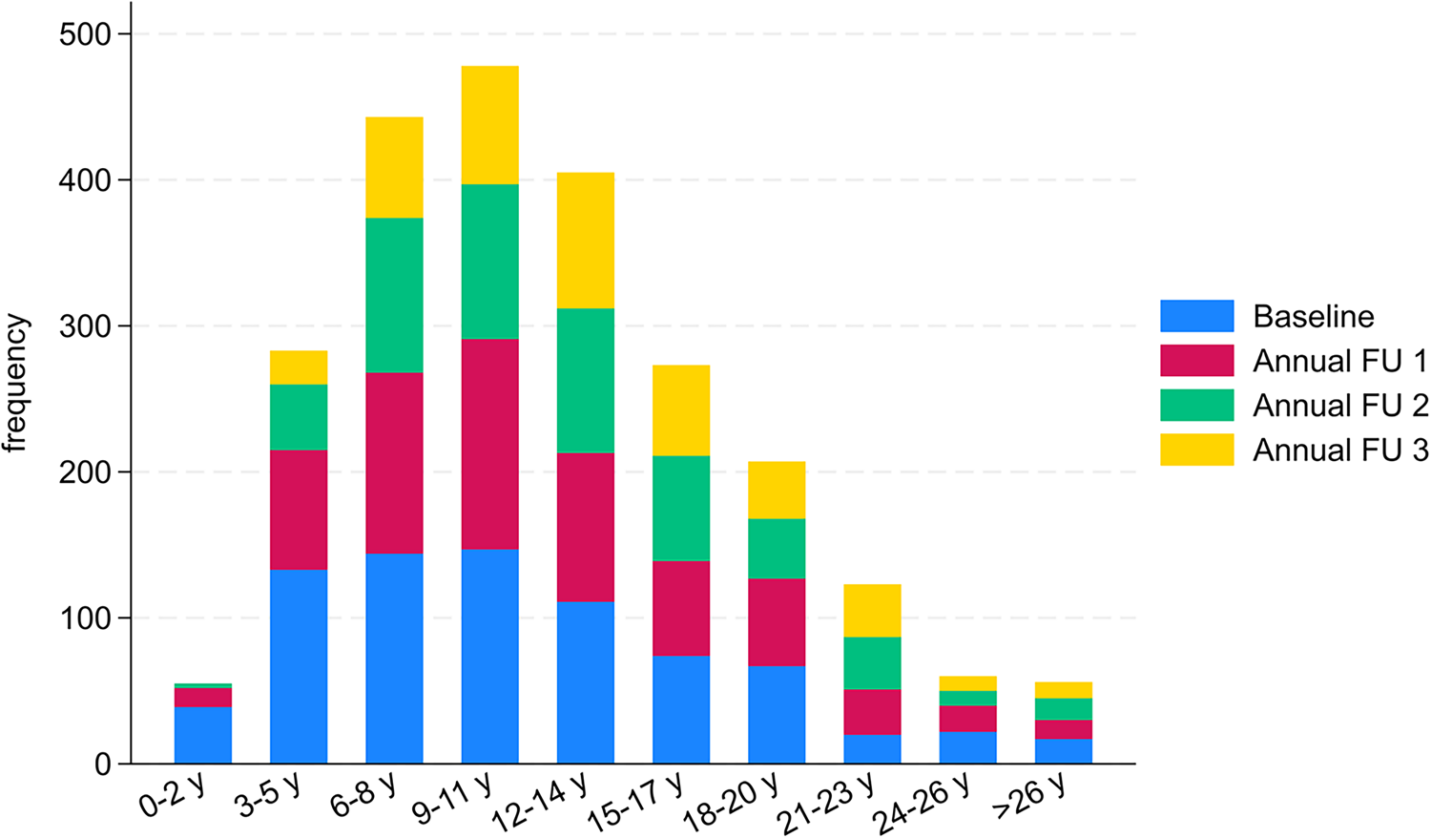
Participants per disease duration group across all visits

SCA patients (*N* = 774) ranged in age from 14 to 85 years (*M* = 50.8, SD = 13.4) at baseline. 48.6% (n = 376) were female. Disease onset was significantly later in SCA 6 patients (*M* = 54.3 years) than in SCA1—3 patients (M = 35.2—38.6 years). A breakdown of the participant flow per disease duration group for each visit is shown in Fig. 1. The mean SARA, EQ-index and PHQ-9 score was 14.83 ± 8.25, 0.64 ± 0.20 and 6.37 ± 5.57, respectively. The large standard deviation of the PHQ-9 score indicates a substantial score variability. However, most participants were not clinically depressive (n _PHQ-9 < 9_ = 77%; see Supplementary Table 2 for severity level distribution of SARA and PHQ-9 at baseline). Detailed information about each SCA subtype’s sociodemographic and clinical description can be found in Table 1.

**Table 1:** Sample characteristics at baseline

Variables	Total	SCA 1	SCA 2	SCA 3	SCA 6	*p value*
*Sex*						
Male n (%)	398 (51.42)	71 (9.17)	74 (9.56)	195 (25.19)	58 (7.49)	0.082
Female n (%)	376 (48.58)	46 (5.94)	88 (11.37)	193 (24.94)	49 (6.33)	
*Age*						
M ± SD	50.77 ± 13.38	46.33 ± 12.25	46.36 ± 13.34	50.02 ± 11.64	64.99 ± 10.92	** > 0.001**
Min—max	14–85	18–76	18–84	14–81	37–85	
n	774	117	162	388	107	
Age of onset*						
M ± SD	39.80 ± 12.50	36.86 ± 10.43	35.21 ± 12.46	38.60 ± 10.62	54.32 ± 10.63	** > 0.001**
Min—max	5–77	15–60	7–66	5–76	16–77	
n	774	117	162	388	107	
*Disease duration*						
M ± SD	10.96 ± 6.49	9.47 ± 5.66	11.15 ± 6.05	11.42 ± 6.75	10.67 ± 6.87	**0.031**
Min—max	0–39	0–28	0–30	0–39	1–33	
Q1—Q3 (IQR)	6–15 (9)	5–13 (8)	7–15 (8)	6–16 (10)	6–13 (7)	
*SARA*						
M ± SD	14.83 ± 8.25	15.58 ± 9.08	15.72 ± 7.98	14.12 ± 8.44	15.21 ± 6.78	**0.044**
Min—max	0–40	2–40	2–39	0–40	1–33	
n	772	117	162	386	107	
*EQ-index (EU)*						
M ± SD	0.64 ± 0.20	0.62 ± 0.20	0.65 ± 0.21	0.64 ± 0.21	0.65 ± 0.17	0.555
Min—max	0.03–0.98	0.03–0.98	0.07–0.98	0.03–0.98	0.21–0.98	
n	768	117	162	382	107	
*EQ-VAS*						
M ± SD	62.38 ± 20.96	59.57 ± 21.88	63.26 ± 20.56	62.46 ± 21.40	63.76 ± 18.90	0.515
Min—max	0–100	0–100	0–100	0–100	20–100	
n	749	112	159	372	106	
*PHQ-9*						
M ± SD	6.37 ± 5.57	6.68 ± 6.25	5.50 ± 4.78	6.94 ± 5.64	5.28 ± 5.40	**0.002**
Min—max	0–27	0–26	0–23	0–27	0–27	
N	758	115	159	377	107	

Descriptive information at baseline for each SCA subtype. *M* mean, *SD* standard deviation, *Min* minimum, *Max* maximum, *SARA* Scale for Assessment and Rating of Ataxia, *EQ-VAS* EuroQol Visual Analog Scale, *PHQ-9* Patient Health Questionnaire. Kruskall–Wallis was the statistical test used for continuous variable and Chi^2^ test of independence for used for the categorical variable. Detailed breakdown of SARA and PHQ-9 subgroups can be found in Supplementary Table 2. *Age of onset was defined by the reported onset of gait disturbances

### The Course of HRQoL and Depressive Symptoms Severity

**Fig. 2:**
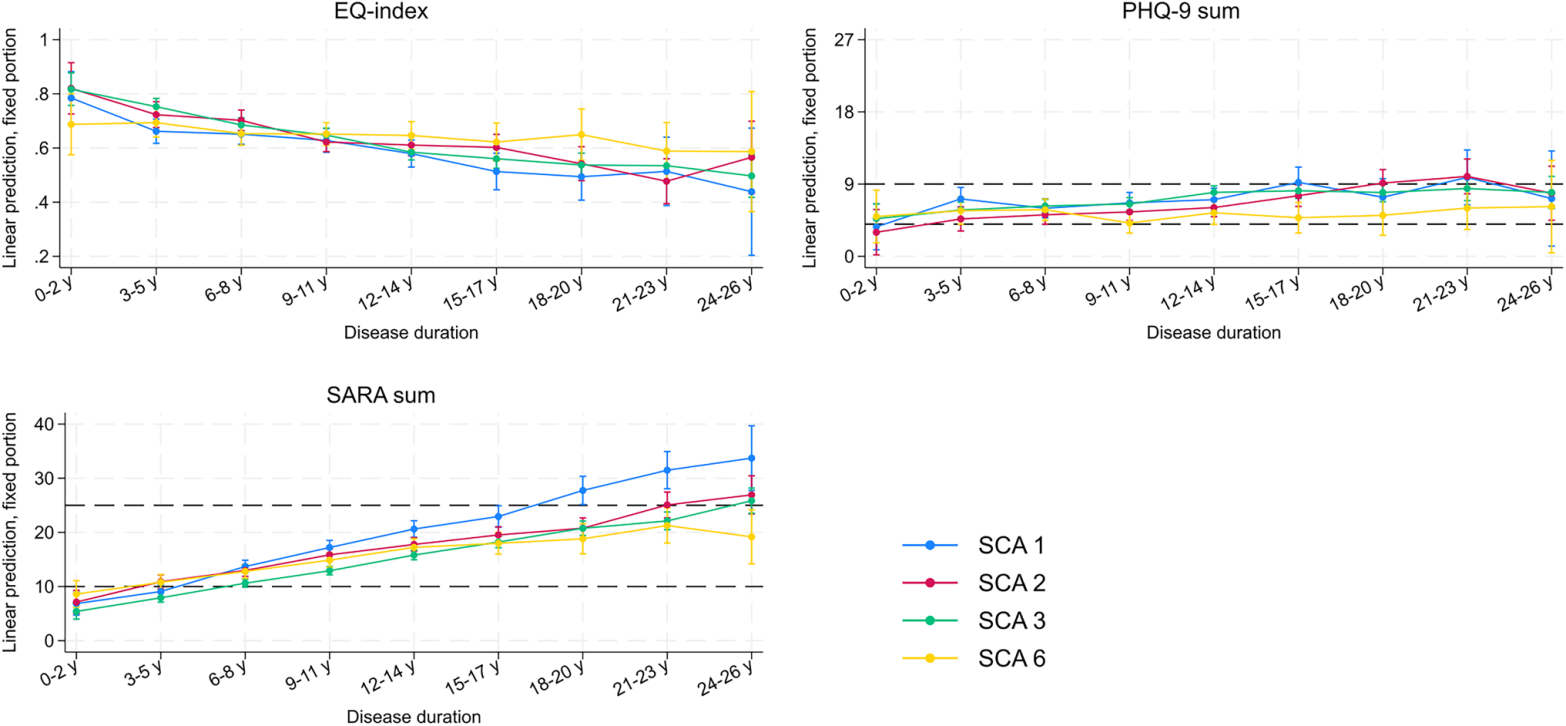
Plotted Estimated Marginal Mean of HRQoL (EQ-5D), depressive symptom severity (PHQ-9) and ataxia severity (SARA) over disease duration. *Note*. SCA subtype was included in the general model to allow for direct comparisons. Age of onset and gender were controlled. Reference lines correspond to the severity cut-off values described in the methods section. EMM (Estimated Marginal Mean) table is available in the Supplementary material

The univariate model shows progressively declining HRQoL (b = − 0.013,* p* < 0.001) and increasing depressive symptom severity (b = 0.157,* p* < 0.001) throughout the disease progression (see Fig. 2). While HRQoL significantly decreased across all SCA subtypes, a significant increase in depressive symptom severity could only be found for SCA 2 and 3 (see Table 3). The estimated course of HRQoL, depressive symptom severity, and ataxia severity across disease duration and SCA type is demonstrated in Fig. 2. In contrast, the marginal effect estimate tables are available in Supplementary Tables 5, 6, 7.

**Fig. 3:**
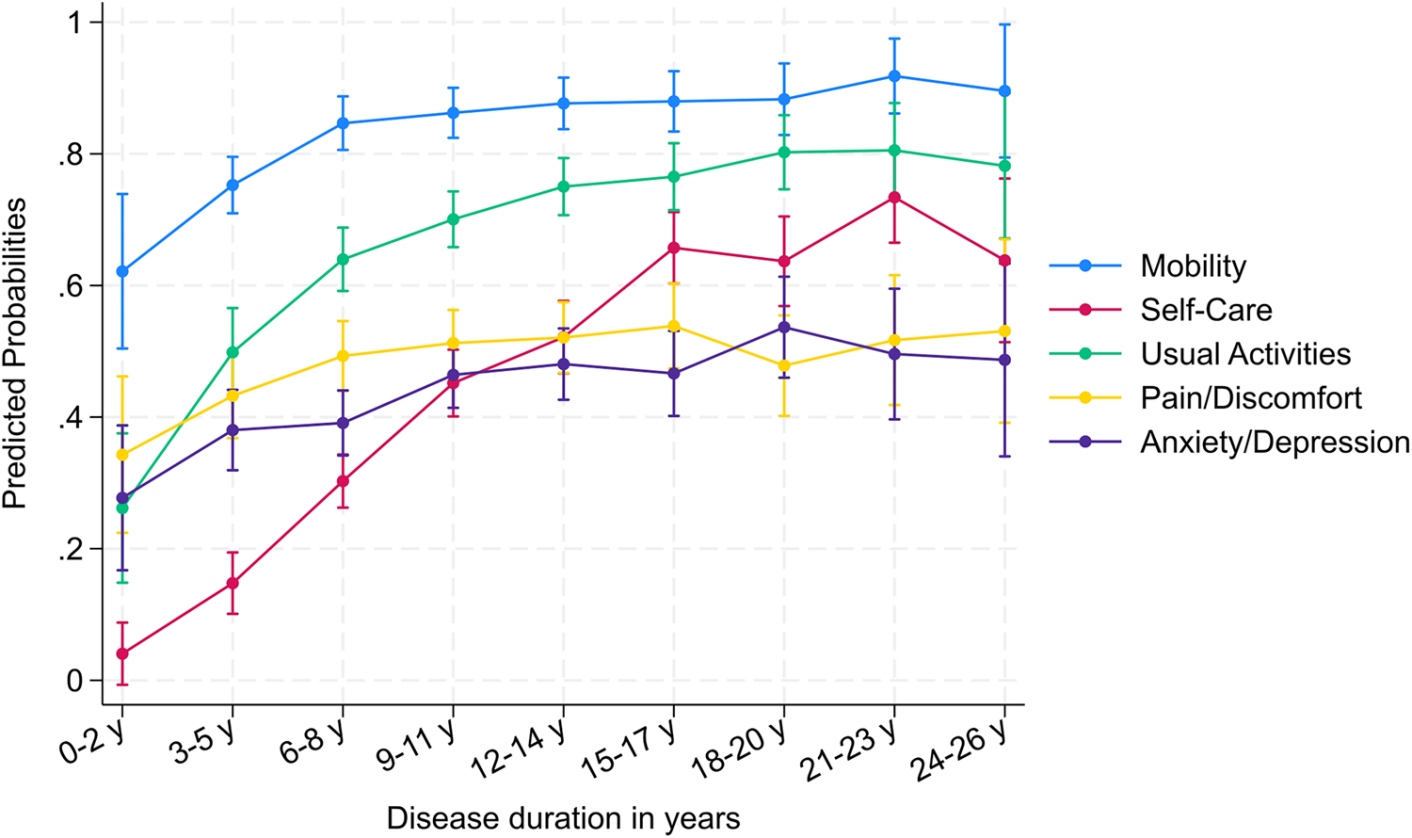
Predicted probability of reporting difficulties in each EQ-subdimension following disease onset. *Note.* The rating 2 & 3 are merged as “problem” and 1 as “no problem”. Only the predicted probability of participants rating “problem” is shown. Self-care shows the fastest rate of change and the largest variability compared to other sub-dimensions. Complete breakdown of each subdimension rating can be observed in Supplementary Fig. 2

The estimated marginal effect on the EQ-5D subdimensions over disease duration (see Supplementary Table 9) revealed that patients were most likely affected by mobility problems (62.15%, CI 50.41–73.89) at disease onset, followed by having pain or feeling discomfort (34.29%, CI 22.41–46.17), being anxious or depressed (27.73%, CI 16.72–38.73), and problems carrying out usual activities (26.18%, CI 14.83–37.54) (see also Supplementary Fig. 2). Problems in self-care were the least prevalent at disease onset (0.04%, CI − 0.68 to 8.78) but had the highest likelihood to progress (OR 1.32, CI 1.25–1.39, *p* < 0.001) with the disease progression, especially during the first 15 years of the disease. Compared to the trajectory of self-care, mobility problems (OR 1.15, CI 1.10–1.22, *p* < 0.001) and problems in usual activities (OR 1.16, CI 1.12–1.21, *p* < 0.001), the dimensions pain/discomfort (OR 1.05, CI 1.01–1.08, *p* < 0.001) and anxiety/depression (OR 1.07, CI 1.04–1.10, *p* < 0.001) progressed less intensively over time (see Table 2 & Fig. 3). Twenty-six years since onset, most participants, regardless of SCA subtype, experienced moderate levels of impairment in all aspects of HRQoL.

**Table 2:** Summarized results of mixed-effect ordinal logistic regressions of each EQ-5D subdimension using the complete cases

*Disease duration* *(fixed effect estimates)*	Odds ratio	95% CI	*p* value
Mobility	1.153	[1.091, 1.219]	0.000***
Self-care	1.316	[1.251, 1.385]	0.000***
Usual activities	1.162	[1.120, 1.207]	0.000***
Pain/discomfort	1.045	[1.014, 1.077]	0.004***
Anxiety/depression	1.065	[1.033, 1.099]	0.000***

Disease duration *(participants: random intercept)*	Coeff. (Log Odds)	95% CI	ICC (%)
Mobility	11.655	[7.442, 18.254]	77.987
Self-care	9.083	[6.700, 12.313]	73.411
Usual activities	5.869	[4.401, 7.826]	64.079
Pain/discomfort	5.032	[3.895, 6.500]	60.466
Anxiety/depression	5.268	[4.071, 6.817]	61.559

N_obs_ = 2,376 (n_group_ = 768). This table summarizes results from five separate ordinal mixed-effects regressions, each examining the relationship between disease duration and an EQ-5D subdimension. The random effect coefficients reflect variability between participants in each subdimension. The ICC quantifies the proportion of total variance attributable to differences between participants, indicating the extent to which individual-specific factors influence the outcome. A high ICC highlights substantial between-participant variability, suggesting that individual-level characteristics could play a significant role

### Associated factors of HRQoL and depressive symptoms severity

As expected, the multivariate model shows that ataxia severity was associated with more decline in HRQoL (b = − 0.012, *p* < 0.001) and depressive symptom severity (b = 0.083, *p* < 0.001). While this effect persisted throughout all SCA subtypes for HRQoL, an impact of ataxia severity on the course of depression was only seen for SCA 1 (b = 0.127, *p* < 0.05) and 3 (b = 0.122, *p* < 0.001). Gender mainly had no effect or interaction effect on HRQoL at any level of the disease duration in all SCA subtypes, except for a higher decrease in HRQoL in female SCA 6 patients (b = − 0.037,* p* < 0.05). However, female patients have a higher average of PHQ-9 sum scores over time across all SCA subtypes (b = 0.609,* p* < 0.01), predominantly female SCA 1 patients (b = 1.564,* p* < 0.05) (see Table 3). A later onset in SCA 3 was also generally associated with a lower average PHQ-9 sum score throughout the disease duration than earlier onset (b = − 0.072,* p* < 0.05).

**Table 3:** Prediction factors of HRQoL and depression across SCA subtype from bivariate (disease duration as sole predictor) and multivariate models (various predictors)

*Bivariate models* *beta coefficients*	Total	SCA 1	SCA 2	SCA 3	SCA 6
*HRQoL* *(EQ-5D-3L)*					
Disease duration	− 0.013***	− 0.014***	− 0.012***	− 0.016***	− 0.004*
Intercept	0.775***	0.751***	0.776***	0.813***	0.690***
*Depressive symptoms (PHQ-9)*					
Disease duration	0.157***	0.156	0.245***	0.175***	-0.036
Intercept	4.826***	5.267***	3.437***	5.042***	5.570***
*Multivariate models*					
*HRQoL* *(EQ-5D-3L)*					
Disease duration	− 0.004*	− 0.002	− 0.006	− 0.003	0.006
Disease onset	− 0.001	0.001	− 0.003	0.001	0.003
Duration*onset	0.000	− 0.000	0.000	− 0.000	− 0.000
Ataxia severity (SARA)	− 0.012***	− 0.010***	− 0.014***	− 0.012***	− 0.010***
Depression (PHQ-9)	− 0.012***	− 0.011***	− 0.012***	− 0.011***	− 0.012***
Female sex	− 0.015	0.006	− 0.028	− 0.013	− 0.037*
Intercept	0.938***	0.855***	1.079***	0.895***	0.697***
*Depressive symptoms (PHQ-9)*					
Disease duration	0.004	− 0.560	0.146	− 0.012	− 0.282
Disease onset	− 0.029	− 0.099	0.055	− 0.072*	− 0.077
Duration*onset	-− 0.000	0.014	− 0.002	− 0.001	0.004
Ataxia severity (SARA)	0.083***	0.127*	0.034	0.122***	0.085
Female sex	0.609**	1.564*	− 0.119	0.516	1.001
Mobility (EQ-5D)	0.460	0.906	0.956	0.578	− 0.647
Self-care (EQ-5D)	0.509*	− 0.219	1.004*	0.562	0.589
Usual activ. (EQ-5D)	1.014***	0.760	0.777	1.159***	0.717
Pain/discomf. (EQ-5D)	1.012***	2.294***	0.462	0.644*	0.639
Anxiety/depr. (EQ-5D)	3.089***	2.513***	3.522***	3.029***	3.561***
Intercept	2.996**	4.504	-0.799	5.158***	6.526

^*^p < 0.05, **p < 0.01, ***p < 0.001

*Bivariate model for HRQoL:* For Total: N_groups_ = 768; N_obs_ = 2,376; Marginal R^2^ = 14.76%; Conditional R^2^ = 65.46%; ICC = 38.782%; AIC = -1,922.907; BIC = -1,894.041; *Bivariate model for* PHQ-9**:** For Total: N_groups_ = 762; N_obs_ = 2,322; Marginal R^2^ = 2.95%; Conditional R^2^ = 64.50%; ICC = 57.653%; AIC = 13,611.41; BIC = 13,640.16; *Multivariate model for HRQoL:* For Total: N_groups_ = 761; N_obs_ = 2,310; Marginal R^2^ = 47.09%; Conditional R^2^ = 67.84%; ICC = 30.017%; AIC = -2,856.725; BIC = -2,799.275.; *Multivariate model for PHQ-9*: For Total: N_groups_ = 761; N_obs_ = 2,310; Marginal R^2^ = 21.33%; Conditional R^2^ = 61.96%; ICC = 45.226%; AIC = 13,166.71; BIC = 13,247.14; variance components are not shown in the table

Concerning the interaction of HRQoL dimensions and depressive symptoms, aside from the significantly negative effect of depressive symptoms severity on HRQoL (b = − 0.012, *p* < 0.001), holding EQ-5D sub-dimensions constant on the depressive symptom severity trajectory revealed that mobility problems were not a significant predictor of the increase in depressive symptoms severity. However, problems in carrying out usual activities (b = 1.014, *p* < 0.001), having pain or feeling discomfort (b = 1.012, *p* < 0.001), difficulties in caring for oneself (b = 0.505, *p* < 0.05), and being anxious (b = 3.089, *p* < 0.001) dimensions of EQ-5D proved to be significant predictors. While anxiety consistently predicts the increase of depressive symptoms across all subtypes, having pain or feeling discomfort, problems in self-care and in carrying out usual activities predicted the increase in SCA 1, 2 and 3 with different intensities.

Other accompanying symptoms, such as speech disturbances, which often develop as the disease progresses, may help explain the flatter anxiety/depression trajectory. An additional exploratory mixed-effects model examined speech disturbance levels in SARA (grouped into three categories beyond the normal rating of 0) and their relationship with depression, as indicated by a PHQ-9 score greater than 9 (clinically depressed). The analysis found that individuals with higher levels of speech disturbance had a greater probability of being depressed on average. Specifically, speech disturbance levels 1–2 were associated with an odds ratio (OR) of 1.22 (95% CI 0.45–1.99, *p* < 0.01), levels 3–4 with an OR of 1.83 (95% CI 0.99–2.67, *p* < 0.001), and levels 5–6 with an OR of 2.85 (95% CI 1.54–4.17, *p* < 0.001). See Supplementary Table 4 for the detailed breakdown of speech disturbance levels.

## Discussion

### Summary of findings

This paper aimed to demonstrate the course of HRQoL and depressive symptoms from disease onset over a long observational period of up to 26 years of disease duration in the most common SCAs (namely SCA1, 2, 3 and 6) and explored potential influencing factors. HRQoL consistently and intensively decreased throughout disease progression, primarily driven by ataxia and depressive symptoms and exacerbated by increasing difficulties with self-care, usual activities, and mobility. While mobility problems were already highly prevalent at disease onset, difficulties with self-care and usual activities were less prevalent at the onset but were increasingly reported within the first 10 to 15 years after onset.

On the other hand, depressive symptoms severity was not as pronounced as HRQoL throughout the course of the disease and did not consistently increase across SCA types, especially not in SCA 6. However, female gender, ataxia severity, anxiety, pain, and problems with usual activities and self-care, but not mobility restrictions, were associated with higher depressive symptoms over time. Notably, later onset is associated with a lower average of depressive symptoms severity in SCA 3. Still, the predicted trajectory did not cross the threshold of clinical depression.

### The course of HRQoL and depressive symptoms

There is limited evidence about the course of HRQoL and depressive symptoms severity as well as findings regarding the course of HRQoL domains and overall HRQoL across disease duration in ataxia diseases. Therefore, our results indicated, for the first time, how HRQoL dimensions, as well as depressive symptoms severity, evolve. Underlining differential needs in each disease stage, our HRQoL subdimension analyses revealed the steepest slope for self-care limitations. The likelihood of experiencing self-care problems increases significantly at two points: moderate issues become more common around 14 years after onset, while extreme problems peak around 22 years. Mobility restrictions and limitations, however, are already widespread at disease onset, with approximately 65% reporting moderate issues compared to 35% with no problems. (see Supplementary Fig. 1B)

In addition, limitations in usual activities rise sharply between 3 and 17 years after onset (see Supplementary Fig. 1C). This could reflect the variations in cognitive and physical abilities and emotional well-being throughout the disease duration, influencing the participants’ capacity for self-care and maintaining routine. (see also Rockwood et al., 2014 [34]).

In contrast, the HRQoL subdimensions, pain/discomfort and anxiety/depression (EQ-5D), and the depressive symptom severity (PHQ-9) were relatively stable over the observed disease course. This could be due to the undervaluation or ambiguous interpretation of composite items of the EQ-5D [35, 36], even though several studies already underline the validity of the EQ-5D in ataxia diseases [37, 38]. Alternatively, the lack of granularity in the EQ-5D-3L descriptive system could not accurately represent the participant’s circumstances [39, 40], especially as seen in the mobility item where the most severe option is labeled as “confined to bed”, which does not fit the situation of SCA patients and could have caused the low probability of selecting this level throughout the disease progression. This partly contradicts the previous study conclusions, in which the physical or mobility-related determinant of HRQoL in SCA patients always had the highest limitations [16].

However, the trend of the PHQ-9 score was in line with the anxiety/depression item of the EQ-5D-3L, indicating a marginal increase and that most patients remained clinically non-depressed (PHQ-9 score < 9) across 26 years after onset. With the current data, we were unable to explain the source of this resilience despite the reportedly relatively high (26%) prevalence of depression as a comorbidity in SCA in other studies [18].

Also, the flatter progression of HRQoL and depressive symptoms in SCA 6 aligns with the slower SCA 6 disease progression indicated in the literature [10, 11, 26]. Surprisingly, depressive symptoms have an opposite trend in SCA 6, where patients get slightly less depressed as the diseases progress, even though this was not significant. One possible explanation is the older average onset age. SCA 6 patients had the oldest average onset age. Older onset is also associated with lower PHQ-9 scores on average in SCA 3. The mild trend in the HRQoL trajectory may suggest a shift in priorities in the older age group, focusing on comfort and care rather than active engagement. This may confirm that the limitations in physical activity in younger individuals and, thereby, the failure of goal pursuit may lead to a steeper decline in HRQoL, as suggested by Allegria [22]. It also highlights the importance of tailored intervention between different SCA types and age groups, as each life phase comes with unique challenges in developmental tasks [41] that possibly result in different coping mechanisms [42].

Also, there was a slightly positive and significant association between age of onset and HRQoL, suggesting that older age may indeed be related to higher HRQoL in SCA 6. However, our study used 4 years of follow-up data with a reshaping of this data across 26 years of disease duration, which limits the generalizability of the presented results. Therefore, further research with more extended observation periods would be needed to explain better this mild trend of HRQoL and depressive symptoms in SCA 6.

### Associated factors of HRQoL

In terms of moderating factors, several cross-sectional studies revealed that lower HRQoL was associated with higher ataxia severity [13, 43–46]. In addition, further associated factors were disease onset, pain, female gender, disease duration and sleep quality [13, 17, 43–48]. While we were unable to evaluate the impact of sleep, our results have demonstrated how female gender, ataxia severity, disease duration, pain and age of onset may differentially affect the course of HRQoL and depressive symptoms of different SCA subtypes within 26 years after onset. In addition, taking all HRQoL sub-dimensions into account, we found that limitations in self-care and usual activities affected patients’ depressive symptom severity. Our results revealed that each SCA subtype has had different predictors for HRQoL and depressive symptoms, even though most patients were not clinically depressed (PHQ-9 score > 9) 26 years after SCA onset. This further highlights the heterogeneity of the symptoms of each subtype and its interaction with each patient’s personal context, e.g., living arrangement, personal circumstances, and life situation in general. Future studies for tailored intervention for each SCA type would need a specific focus on different limitations, such as maintaining self-care, usual activities, managing pain and discomfort, and preventing anxiety and depression. Further explorative analyses, including speech disturbance, as measured by SARA (see Supplementary Table 2), may hint at the significant role in maintaining autonomy among patients with neurodegenerative disorders, as most patients have intact speech. Effective communication with caregivers is essential for expressing needs and preferences [49, 50], which can mitigate feelings of helplessness and dependence. Consequently, patients with intact or minimally impaired speech are likely to experience greater autonomy, reducing the risk of depression despite their physical restrictions. However, more detailed speech assessments should be evaluated in future research.

The captured heterogeneity and participants' resilience may suggest unseen variables explaining the relationship between disease duration, deterioration in HRQoL, and depressive and clinical symptoms. Depression is indeed the most prevalent psychiatric comorbidity in neurodegenerative diseases, including SCA [19, 51]. However, this sample seems to be largely unaffected at baseline (*n* = 584 (77.04%) below 9) with a slow estimated rate of increase of depressive symptoms severity each year (b = 0.143, *p* < 0.001). While this sample generally demonstrated resilience, it is crucial to address the specific needs of more vulnerable patients, as indicated by the multivariate PHQ-9 model: maintaining usual activities for SCA 3 patients, addressing potential depression in those with early disease onset, and pain management for SCA 1 and 3 patients, as well as maintaining self-care capability in SCA 2 patients. Caregivers must be aware of these aspects to ensure a better quality of life and provide more individualized treatment. There might be a need to collect more psychosocial determinants as the physical domains were not consistent determinants of depressive symptoms severity. Despite these findings, the results of this study can provide valuable insights to tailor care for individual SCA patients at specific stages of disease progression. By focusing on maintaining HRQoL and mitigating depressive symptoms, we can significantly improve the quality of life for these patients. Specifically, addressing HRQoL dimensions such as self-care, usual activities, and pain management can substantially impact. To this end, maintaining functionality through physical therapy, assistive devices, and consistent daily routines is crucial throughout the disease’s progression.

While the essentially unchanged level of depression might suggest that these aspects are less influenced by disease duration, it is crucial to recognize that patient perceptions and interpretations of these symptoms may vary widely. In this regard, the role of formal and informal caregivers might be another essential aspect. Sánchez-Lopez et al. [17] demonstrated that the presence of a caregiver accounts for most of the total variance of HRQoL. Informal caregivers provide crucial support, helping patients maintain their daily routines and self-care, which can significantly impact their quality of life and mental health [52–54]. Moreover, studies have shown that interventions supporting caregivers can improve patient well-being [55], strengthening this hypothesis. Conversely, a patient’s declining health can increase their dependence on the caregiver, leading to caregiver burden and potentially worsening the caregiver’s mental health [56], creating a reciprocal relationship where each person’s well-being can significantly impact the other. Future research should focus on these interactions, the heterogeneity of symptom manifestation, and the patient’s disease management strategy.

Without curative treatment, the focus lies on symptom management and maintaining HRQoL and depressive symptoms severity. Identifying and addressing unmet needs due to increasing limitations in self-care, usual activities and mobility with effective communication, e.g., patient involvement in treatment-, care- and support-related decisions, might be vital to maintaining the patient’s autonomy [57, 58]. In light of this, focusing on non-motor symptoms might also be worth it, as maintaining speech ability has increased the HRQoL of SCA patients [59]. Furthermore, mobility-assisted technology and better access to the social care system could reinforce support for self-care and other usual activities for SCA patients. These results further highlight the importance of PROMs (Patient-Reported Outcome Measures) in complementing neurologic measures, as suggested by Jacobi et al.[14], to provide a full picture of a patient’s health status. While Schmahmann’s 2021 PROM for Ataxia [60] is a valuable tool, including HRQoL would offer a more comprehensive understanding of a patient’s health status, especially within longitudinal studies.

### Limitation

Due to attrition, our analysis was limited to baseline and the first three follow-ups, allowing us to study short-term observations of up to 4 years. These observations were scattered across the disease course, encompassing patients with disease durations of up to 26 years. However, this limited dataset did not allow for a comprehensive study of intraindividual long-term trajectories. However, we tried to overcome this limitation by implementing a mixed-effect model that accounts for the dependency between patient ratings. In addition, the focus of the analyses was limited to the 26 years after onset, primarily on SCA 3 due to participant distribution. These factors might also restrict the generalization of the results as the progression of each SCA subtype differs in speed. Interpretation must be exercised with caution because each rating has considerable individual variability, especially regarding PHQ-9 sum scores.

In addition, our cohorts might represent a mentally resilient sample. This bias could be because depressed individuals might be less inclined to participate in the study or are mostly receiving treatment for diagnosed affective disorder. Information regarding medication and other treatments were unfortunately not included in the variable list. Further analyses of the protective factors or other associated factors such as the effect of sleep quality were not possible due to the focus on clinical variables. Particularly sleep quality is often related to psychologic disorders as well as SCA and its accompanying comorbidities. Thereby, sleep quality should be investigated in future studies. Further, the speech disturbance item in SARA shows promising future direction in which most participants can still communicate intelligibly with the caregivers in expressing their needs despite other physical restrictions and how this affects HRQoL in the long term.

## Electronic supplementary material

Below is the link to the electronic supplementary material.

Supplementary file1 (DOCX 784 KB)

Supplementary file2 (TIFF 3484 KB)

Supplementary file3 (TIFF 4281 KB)

Supplementary file4 (TIFF 4281 KB)

## Appendix

Members of the ESMI study group: Aachen: Kathrin Reetz (JARA-Brain Institute, RWTH Aachen University). Azores: Mafalda Raponso (Universidade do Porto), Carlos Gonzales (Centro de Terapia Familiar e Intervenção Sistémica). Bonn: Berkan Koyak, Demet Oender (DZNE Bonn, University of Bonn). Coimbra: Luís Pereira de Almeida, Patrick Silva (GeneT, University of Coimbra), Joana Afonso Ribeiro (Coimbra’s Hospital and University Center). Essen: Andreas Thieme, Friedrich Erdlenbruch (Essen University Hospital). Groenigen: Jeroen de Vries (University Medical Center Gronigen). Baltimore: Chiadikaobi Onyike (John-Hopkins University). London: Paola Giunti, Hector Garcia-Moreno (University College London). Minneapolis: Gülin Öz (University of Minnesota). München: Almut Turid Bischoff (Friedrich Baur Institute, LMU Munich). Nijmegen: Bart van de Warrenburg (Donders Institute for Brain, Cognition, and Behavior, Radboud University Medical Center)., Judith van Gaalen (Rijnstate Hospital). Santander: Jon Infante, Leire Manrique (University Hospital Marqués de Valdecilla-IDIVAL, CIBERNED). Tübingen: Ludger Schöls, Olaf Riess (University of Tübingen).

Members of the EUROSCA study group: Sophie Tezenas du Montcel(UPMC Univ Paris), Peter Bauer (University of Tübingen), Paola Giunti, Arron Cook, Robyn Labrum, Michael H. Parkinson (University College London), Alexandra Durr, Alexis Brice, Perrine Charles (ICM Paris, INSERM Hôpital de la Pitié-Salpêtrière), Cecilia Marelli, Caterina Mariotti, Lorenzo Nanetti, Marta Panzeri (SOSD Genetics of Neurodegenerative and Metabolic Diseases Milan), Maria Rakowicz, Anna Sulek, Anna Sobanska (Institute of Psychiatry and Neurology Warsaw), Holger Hengel (University of Tübingen), Laszlo Baliko (Zala County Hospital), Bela Melegh (University of Pécs), Alessandro Filla, Antonella Antenora (University Naples), José Berciano (University Hospital Marqués de Valdecilla-IDIVAL), Dagmar Timmann (Essen University Hospital), Sandra Szymanski (University Hospital of Bochum), Sylvia Boesch (Innsbruck Medical University), Jun-Suk Kang (University of Frankfurt), Massimo Padolfo (Université Libre de Bruxelles), Jörg B. Schulz (RWTH Aachen University), Sonia Molho (Groupe Hospitalier Pitié-Salpêtrière), Alhassane Diallo (Sorbonne Universités, Université Pierre et Marie Curie).
