# Supplementary material for: Longitudinal description of health-related quality of life and depressive symptoms in polyQ spinocerebellar ataxia patients
Source: J Neurol. 2025 Apr 9;272(5):323. doi: 10.1007/s00415-025-13024-0 (PMC11982069; doi:10.1007/s00415-025-13024-0)
Supplement: Supplementary file 1 — Supplementary file1 (DOCX 784 KB) [file 415_2025_13024_MOESM1_ESM.docx]

# **Supplementaries**

## **Supplementary Table 1**. Onset age percentiles of each SCA sub-type

|  | n | Mean | SD | Min | Max |
| --- | --- | --- | --- | --- | --- |
| **SCA 1** |  |  |  |  |  |
| Early | 42 | 25.55 | 4.60 | 15 | 32 |
| Mid | 41 | 38.15 | 2.98 | 33 | 43 |
| Late | 34 | 49.29 | 4.62 | 44 | 60 |
| **SCA 2** |  |  |  |  |  |
| Early | 57 | 21.75 | 5.17 | 7 | 29 |
| Mid | 56 | 36.02 | 3.27 | 30 | 40 |
| Late | 49 | 49.94 | 6.30 | 41 | 66 |
| **SCA 3** |  |  |  |  |  |
| Early | 137 | 27.61 | 5.20 | 5 | 34 |
| Mid | 129 | 38.61 | 2.35 | 35 | 43 |
| Late | 122 | 50.93 | 5.84 | 44 | 76 |
| **SCA 6** |  |  |  |  |  |
| Early | 43 | 43.95 | 7.52 | 16 | 52 |
| Mid | 36 | 57.44 | 2.65 | 53 | 61 |
| Late | 28 | 66.21 | 3.97 | 62 | 77 |
| Total |  |  |  |  |  |
| Early | 279 | 28.62 | 8.84 | 5 | 52 |
| Mid | 262 | 40.57 | 7.33 | 30 | 61 |
| Late | 233 | 52.32 | 7.60 | 41 | 77 |

*Note*. Results were derived from three equal part percentiles of each SCA sub-type at baseline

## **Supplementary Table 2**. PHQ-9 and SARA sum score distribution at baseline

|  | **SCA 1** | **SCA 2** | **SCA 3** | **SCA 6** | **Total** |
| --- | --- | --- | --- | --- | --- |
| n | 177 (15.10%) | 162 (20.9%) | 388 (50.1%) | 107 (13.8%) | 774 (100.0%) |
| **SARA** |  |  |  |  |  |
| *Mild < 10* | 34 (29.10%) | 39 (24.1%) | 135 (35.0%) | 20 (18.7%) | 228 (29.5%) |
| *Moderate 10 – 25* | 65 (55.60%) | 98 (60.5%) | 205 (53.1%) | 76 (71.0%) | 444 (57.5%) |
| *Advanced > 25* | 18 (15.40%) | 25 (15.4%) | 46 (11.9%) | 11 (10.3%) | 100 (13.0%) |
| **PHQ** |  |  |  |  |  |
| *None 0 – 4* | 55 (47.80%) | 77 (48.4%) | 152 (40.3%) | 62 (57.9%) | 346 (45.6%) |
| *Mild 5 – 9* | 33 (28.70%) | 52 (32.7%) | 126 (33.4%) | 27 (25.2%) | 238 (31.4%) |
| *Moderate 10 – 14* | 13 (11.30%) | 18 (11.3%) | 60 (15.9%) | 10 (9.3%) | 101 (13.3%) |
| *Severe ≥ 15* | 14 (12.20%) | 12 (7.5%) | 39 (10.3%) | 8 (7.5%) | 73 (9.6%) |

## **Supplementary Table 3**. Median rating of each SARA subdimension

| **SARA Category** | **Baseline** | **Follow Up 1** | **Follow Up 2** | **Follow Up 3** |
| --- | --- | --- | --- | --- |
| Gait (0-8) | 3 | 3 | 4 | 4 |
| Stance (0-6) | 2 | 2 | 3 | 3 |
| Sitting (0-4) | 0 | 1 | 2 | 1 |
| Speech  disturbance (0-6) | 2 | 2 | 3 | 2 |
| Finger chase |  |  |  |  |
| ‍ Left (0-4) | 1 | 1 | 1 | 2 |
| ‍ Right (0-4) | 1 | 1 | 1 | 1 |
| Nose-finger |  |  |  |  |
| Left (0-4) | 1 | 1 | 1 | 1 |
| Right (0-4) | 1 | 1 | 1 | 1 |
| Fast alternating hand movement |  |  |  |  |
| Left (0-4) | 1 | 2 | 2 | 2 |
| Right (0-4) | 1 | 1 | 2 | 2 |
| Heel-shin slide |  |  |  |  |
| Left (0-4) | 2 | 2 | 2 | 2 |
| Right (0-4) | 2 | 2 | 2 | 2 |

## **Supplementary Table 4.** Rating distribution of speech disturbance items in SARA

| Speech disturbance | Baseline | Follow up 1 | Follow up 2 | Follow up 3 | Total | % | Cumulative % |
| --- | --- | --- | --- | --- | --- | --- | --- |
| 0 normal | 85 | 64 | 39 | 21 | 209 | 9.5 | 9.5 |
| 1 suggestion of speech disturbance | 188 | 127 | 88 | 62 | 465 | 21.14 | 30.64 |
| 2 impaired but easy to understand | 229 | 205 | 193 | 144 | 771 | 35.05 | 65.69 |
| 3 occasional words difficult to understand | 135 | 120 | 113 | 112 | 480 | 21.82 | 87.51 |
| 4 many words difficult to understand | 55 | 51 | 44 | 47 | 197 | 8.95 | 96.46 |
| 5 only single words understandable | 19 | 18 | 13 | 12 | 62 | 2.82 | 99.28 |
| 6 speech unintelligible/anarthria | 7 | 4 | 3 | 2 | 16 | 0.73 | 100.00 |
| Total | 718 | 589 | 493 | 400 | 2,200 |  |  |

## **Supplementary Table 5.** Estimated marginal mean of PHQ-9 sum score for each SCA subtype over disease duration

| SCA subtype*DD | Margin | Std. err. | z | P>z | [95% conf. | interval] |
| --- | --- | --- | --- | --- | --- | --- |
| SCA 1*0-2 y | 3.677 | 1.464 | 2.510 | 0.012 | 0.807 | 6.547 |
| SCA 1*3-5 y | 7.152 | 0.727 | 9.830 | 0.000 | 5.727 | 8.578 |
| SCA 1*6-8 y | 5.991 | 0.610 | 9.820 | 0.000 | 4.795 | 7.186 |
| SCA 1*9-11 y | 6.665 | 0.659 | 10.110 | 0.000 | 5.373 | 7.956 |
| SCA 1*12-14 y | 7.063 | 0.725 | 9.750 | 0.000 | 5.643 | 8.483 |
| SCA 1*15-17 y | 9.218 | 0.967 | 9.530 | 0.000 | 7.322 | 11.114 |
| SCA 1*18-20 y | 7.364 | 1.178 | 6.250 | 0.000 | 5.055 | 9.672 |
| SCA 1*21-23 y | 9.831 | 1.748 | 5.630 | 0.000 | 6.406 | 13.256 |
| SCA 1*24-26 y | 7.199 | 3.026 | 2.380 | 0.017 | 1.268 | 13.131 |
|  |  |  |  |  |  |  |
| SCA 2*0-2 y | 2.999 | 1.445 | 2.080 | 0.038 | 0.167 | 5.830 |
| SCA 2*3-5 y | 4.657 | 0.769 | 6.060 | 0.000 | 3.150 | 6.164 |
| SCA 2*6-8 y | 5.176 | 0.605 | 8.560 | 0.000 | 3.990 | 6.361 |
| SCA 2*9-11 y | 5.535 | 0.539 | 10.260 | 0.000 | 4.478 | 6.591 |
| SCA 2*12-14 y | 6.078 | 0.581 | 10.470 | 0.000 | 4.940 | 7.216 |
| SCA 2*15-17 y | 7.553 | 0.684 | 11.040 | 0.000 | 6.212 | 8.894 |
| SCA 2*18-20 y | 9.137 | 0.853 | 10.710 | 0.000 | 7.465 | 10.809 |
| SCA 2*21-23 y | 9.959 | 1.105 | 9.010 | 0.000 | 7.793 | 12.126 |
| SCA 2*24-26 y | 7.858 | 1.718 | 4.570 | 0.000 | 4.490 | 11.225 |
|  |  |  |  |  |  |  |
| SCA 3*0-2 y | 4.692 | 0.929 | 5.050 | 0.000 | 2.871 | 6.514 |
| SCA 3*3-5 y | 5.771 | 0.496 | 11.640 | 0.000 | 4.799 | 6.743 |
| SCA 3*6-8 y | 6.278 | 0.409 | 15.350 | 0.000 | 5.476 | 7.080 |
| SCA 3*9-11 y | 6.528 | 0.396 | 16.470 | 0.000 | 5.751 | 7.305 |
| SCA 3*12-14 y | 7.966 | 0.417 | 19.080 | 0.000 | 7.148 | 8.785 |
| SCA 3*15-17 y | 8.175 | 0.493 | 16.590 | 0.000 | 7.209 | 9.141 |
| SCA 3*18-20 y | 7.954 | 0.588 | 13.540 | 0.000 | 6.802 | 9.105 |
| SCA 3*21-23 y | 8.453 | 0.773 | 10.940 | 0.000 | 6.938 | 9.968 |
| SCA 3*24-26 y | 7.952 | 1.023 | 7.770 | 0.000 | 5.947 | 9.958 |
|  |  |  |  |  |  |  |
| SCA 6*0-2 y | 4.960 | 1.673 | 2.960 | 0.003 | 1.681 | 8.240 |
| SCA 6*3-5 y | 5.685 | 0.860 | 6.610 | 0.000 | 3.998 | 7.371 |
| SCA 6*6-8 y | 5.806 | 0.690 | 8.420 | 0.000 | 4.455 | 7.158 |
| SCA 6*9-11 y | 4.148 | 0.642 | 6.470 | 0.000 | 2.891 | 5.406 |
| SCA 6*12-14 y | 5.424 | 0.740 | 7.330 | 0.000 | 3.974 | 6.875 |
| SCA 6*15-17 y | 4.812 | 0.974 | 4.940 | 0.000 | 2.903 | 6.721 |
| SCA 6*18-20 y | 5.109 | 1.271 | 4.020 | 0.000 | 2.617 | 7.601 |
| SCA 6*21-23 y | 6.015 | 1.363 | 4.410 | 0.000 | 3.343 | 8.686 |
| SCA 6*24-26 y | 6.200 | 2.940 | 2.110 | 0.035 | 0.438 | 11.962 |

## **Supplementary Table 6.** Estimated marginal mean of EQ index for each SCA subtype over disease duration

| SCA subtype*DD | Margin | Std. err. | z | P>z | [95% conf. | interval] |
| --- | --- | --- | --- | --- | --- | --- |
| SCA 1*0-2 y | 0.785 | 0.050 | 15.830 | 0.000 | 0.688 | 0.882 |
| SCA 1*3-5 y | 0.662 | 0.023 | 29.050 | 0.000 | 0.617 | 0.707 |
| SCA 1*6-8 y | 0.651 | 0.019 | 33.950 | 0.000 | 0.614 | 0.689 |
| SCA 1*9-11 y | 0.628 | 0.022 | 28.120 | 0.000 | 0.584 | 0.672 |
| SCA 1*12-14 y | 0.579 | 0.025 | 22.820 | 0.000 | 0.529 | 0.629 |
| SCA 1*15-17 y | 0.513 | 0.034 | 14.900 | 0.000 | 0.446 | 0.581 |
| SCA 1*18-20 y | 0.494 | 0.044 | 11.160 | 0.000 | 0.407 | 0.581 |
| SCA 1*21-23 y | 0.514 | 0.064 | 7.980 | 0.000 | 0.388 | 0.640 |
| SCA 1*24-26 y | 0.439 | 0.120 | 3.660 | 0.000 | 0.204 | 0.673 |
|  |  |  |  |  |  |  |
| SCA 2*0-2 y | 0.820 | 0.048 | 16.970 | 0.000 | 0.725 | 0.915 |
| SCA 2*3-5 y | 0.723 | 0.024 | 29.680 | 0.000 | 0.675 | 0.771 |
| SCA 2*6-8 y | 0.702 | 0.019 | 36.140 | 0.000 | 0.664 | 0.740 |
| SCA 2*9-11 y | 0.622 | 0.018 | 34.560 | 0.000 | 0.587 | 0.657 |
| SCA 2*12-14 y | 0.611 | 0.020 | 30.120 | 0.000 | 0.571 | 0.651 |
| SCA 2*15-17 y | 0.602 | 0.024 | 24.670 | 0.000 | 0.555 | 0.650 |
| SCA 2*18-20 y | 0.542 | 0.032 | 16.970 | 0.000 | 0.479 | 0.605 |
| SCA 2*21-23 y | 0.477 | 0.042 | 11.260 | 0.000 | 0.394 | 0.560 |
| SCA 2*24-26 y | 0.566 | 0.068 | 8.330 | 0.000 | 0.433 | 0.699 |
|  |  |  |  |  |  |  |
| SCA 3*0-2 y | 0.817 | 0.031 | 26.730 | 0.000 | 0.757 | 0.877 |
| SCA 3*3-5 y | 0.753 | 0.016 | 48.260 | 0.000 | 0.722 | 0.783 |
| SCA 3*6-8 y | 0.685 | 0.013 | 51.310 | 0.000 | 0.659 | 0.711 |
| SCA 3*9-11 y | 0.647 | 0.013 | 48.660 | 0.000 | 0.621 | 0.673 |
| SCA 3*12-14 y | 0.584 | 0.015 | 39.880 | 0.000 | 0.556 | 0.613 |
| SCA 3*15-17 y | 0.560 | 0.018 | 31.160 | 0.000 | 0.525 | 0.596 |
| SCA 3*18-20 y | 0.537 | 0.022 | 24.240 | 0.000 | 0.494 | 0.581 |
| SCA 3*21-23 y | 0.535 | 0.029 | 18.240 | 0.000 | 0.477 | 0.592 |
| SCA 3*24-26 y | 0.497 | 0.040 | 12.270 | 0.000 | 0.418 | 0.576 |
|  |  |  |  |  |  |  |
| SCA 6*0-2 y | 0.687 | 0.057 | 11.960 | 0.000 | 0.575 | 0.800 |
| SCA 6*3-5 y | 0.694 | 0.027 | 25.430 | 0.000 | 0.640 | 0.747 |
| SCA 6*6-8 y | 0.653 | 0.022 | 29.510 | 0.000 | 0.610 | 0.696 |
| SCA 6*9-11 y | 0.652 | 0.021 | 30.400 | 0.000 | 0.610 | 0.694 |
| SCA 6*12-14 y | 0.646 | 0.026 | 24.750 | 0.000 | 0.595 | 0.698 |
| SCA 6*15-17 y | 0.622 | 0.036 | 17.390 | 0.000 | 0.552 | 0.692 |
| SCA 6*18-20 y | 0.650 | 0.048 | 13.460 | 0.000 | 0.555 | 0.744 |
| SCA 6*21-23 y | 0.588 | 0.054 | 10.910 | 0.000 | 0.483 | 0.694 |
| SCA 6*24-26 y | 0.587 | 0.113 | 5.190 | 0.000 | 0.365 | 0.809 |

## **Supplementary Table 7.** Estimated marginal mean of SARA sum score for each SCA subtype over disease duration

| SCA subtype*DD | Margin | Std. err. | z | P>z | [95% conf. | interval] |
| --- | --- | --- | --- | --- | --- | --- |
| SCA 1*0-2 y | 6.846 | 1.094 | 6.260 | 0.000 | 4.702 | 8.990 |
| SCA 1*3-5 y | 9.087 | 0.629 | 14.450 | 0.000 | 7.855 | 10.319 |
| SCA 1*6-8 y | 13.698 | 0.587 | 23.320 | 0.000 | 12.547 | 14.849 |
| SCA 1*9-11 y | 17.217 | 0.666 | 25.840 | 0.000 | 15.910 | 18.523 |
| SCA 1*12-14 y | 20.620 | 0.775 | 26.600 | 0.000 | 19.101 | 22.139 |
| SCA 1*15-17 y | 22.940 | 1.004 | 22.850 | 0.000 | 20.972 | 24.908 |
| SCA 1*18-20 y | 27.750 | 1.325 | 20.940 | 0.000 | 25.153 | 30.347 |
| SCA 1*21-23 y | 31.491 | 1.755 | 17.940 | 0.000 | 28.051 | 34.932 |
| SCA 1*24-26 y | 33.718 | 3.047 | 11.060 | 0.000 | 27.745 | 39.690 |
|  |  |  |  |  |  |  |
| SCA 2*0-2 y | 7.137 | 1.093 | 6.530 | 0.000 | 4.995 | 9.279 |
| SCA 2*3-5 y | 10.893 | 0.643 | 16.940 | 0.000 | 9.633 | 12.153 |
| SCA 2*6-8 y | 12.961 | 0.558 | 23.210 | 0.000 | 11.866 | 14.055 |
| SCA 2*9-11 y | 15.862 | 0.557 | 28.480 | 0.000 | 14.770 | 16.953 |
| SCA 2*12-14 y | 17.773 | 0.633 | 28.080 | 0.000 | 16.532 | 19.014 |
| SCA 2*15-17 y | 19.529 | 0.758 | 25.750 | 0.000 | 18.043 | 21.016 |
| SCA 2*18-20 y | 20.771 | 0.962 | 21.600 | 0.000 | 18.886 | 22.656 |
| SCA 2*21-23 y | 25.072 | 1.216 | 20.630 | 0.000 | 22.689 | 27.454 |
| SCA 2*24-26 y | 26.933 | 1.793 | 15.020 | 0.000 | 23.418 | 30.448 |
|  |  |  |  |  |  |  |
| SCA 3*0-2 y | 5.413 | 0.731 | 7.410 | 0.000 | 3.980 | 6.845 |
| SCA 3*3-5 y | 7.931 | 0.416 | 19.080 | 0.000 | 7.117 | 8.746 |
| SCA 3*6-8 y | 10.627 | 0.375 | 28.300 | 0.000 | 9.891 | 11.362 |
| SCA 3*9-11 y | 12.917 | 0.393 | 32.900 | 0.000 | 12.147 | 13.686 |
| SCA 3*12-14 y | 15.812 | 0.443 | 35.690 | 0.000 | 14.944 | 16.681 |
| SCA 3*15-17 y | 18.221 | 0.537 | 33.910 | 0.000 | 17.168 | 19.274 |
| SCA 3*18-20 y | 20.759 | 0.677 | 30.670 | 0.000 | 19.432 | 22.085 |
| SCA 3*21-23 y | 22.118 | 0.839 | 26.360 | 0.000 | 20.473 | 23.763 |
| SCA 3*24-26 y | 25.852 | 1.187 | 21.780 | 0.000 | 23.525 | 28.178 |
|  |  |  |  |  |  |  |
| SCA 6*0-2 y | 8.619 | 1.247 | 6.910 | 0.000 | 6.175 | 11.063 |
| 4-6SCA 6*3-5 y | 10.788 | 0.726 | 14.860 | 0.000 | 9.366 | 12.210 |
| SCA 6*6-8 y | 12.798 | 0.647 | 19.790 | 0.000 | 11.530 | 14.065 |
| SCA 6*9-11 y | 14.874 | 0.678 | 21.940 | 0.000 | 13.546 | 16.203 |
| SCA 6*12-14 y | 17.232 | 0.814 | 21.180 | 0.000 | 15.637 | 18.827 |
| SCA 6*15-17 y | 17.987 | 1.021 | 17.620 | 0.000 | 15.987 | 19.987 |
| SCA 6*18-20 y | 18.814 | 1.420 | 13.250 | 0.000 | 16.030 | 21.597 |
| SCA 6*21-23 y | 21.271 | 1.654 | 12.860 | 0.000 | 18.029 | 24.513 |
| SCA 6*24-26 y | 19.181 | 2.553 | 7.510 | 0.000 | 14.178 | 24.184 |

## **Supplementary Table 8**. PHQ-9 model without EQ sub dimension: Anxiety/Depression

| Variable | Total | SCA 1 | SCA 2 | SCA 3 | SCA 6 |
| --- | --- | --- | --- | --- | --- |
| DD | 0.042 | -0.468 | 0.252 | -0.062 | -0.198 |
| Onset age | -0.029 | -0.088 | 0.099 | -0.099* | -0.081 |
|  |  |  |  |  |  |
| DD*Onset age | -0.001 | 0.012 | -0.004 | 0.001 | 0.002 |
|  |  |  |  |  |  |
| Female | 0.897** | 1.722* | 0.271 | 0.699 | 1.721* |
|  |  |  |  |  |  |
| SARA | 0.096*** | 0.120* | 0.075 | 0.133*** | 0.073 |
| MO | 0.541 | 1.255 | 1.067 | 0.661 | -0.909 |
| SC | 0.680** | -0.281 | 1.175* | 0.867** | 0.701 |
| UA | 1.311*** | 1.310 | 0.992 | 1.336*** | 1.283 |
| PD | 1.294*** | 2.475*** | 0.878* | 0.865** | 1.190* |
| Intercept | 3.484** | 4.312 | -2.275 | 6.757*** | 7.809* |

* *p* < 0.05, ** *p* < 0.01, *** *p* < 0.001.

## **Supplementary Table 9**. Estimated Marginal Probabilities of each EQ-5D subdimension

| DD | MO | CI 95% | SC | CI 95% | UA | CI 95% | PD | CI 95% | AD | CI 95% |
| --- | --- | --- | --- | --- | --- | --- | --- | --- | --- | --- |
| 0-2 y | 0.621 | [0.504, 0.739] | 0.040 | [-0.007, 0.088] | 0.262 | [0.148, 0.375] | 0.343 | [0.224, 0.462] | 0.277 | [0.167, 0.387] |
| 3-5 y | 0.753 | [0.710, 0.795] | 0.148 | [0.101, 0.194] | 0.498 | [0.431, 0.566] | 0.432 | [0.368, 0.497] | 0.380 | [0.319, 0.441] |
| 6-8 y | 0.847 | [0.806, 0.887] | 0.303 | [0.262, 0.343] | 0.640 | [0.592, 0.688] | 0.493 | [0.440, 0.546] | 0.391 | [0.342, 0.440] |
| 9-11 y | 0.862 | [0.824, 0.900] | 0.452 | [0.401, 0.502] | 0.700 | [0.658, 0.743] | 0.512 | [0.462, 0.563] | 0.464 | [0.414, 0.514] |
| 12-14 y | 0.877 | [0.838, 0.916] | 0.521 | [0.466, 0.577] | 0.750 | [0.707, 0.794] | 0.521 | [0.467, 0.575] | 0.480 | [0.426, 0.535] |
| 15-17 y | 0.880 | [0.834, 0.925] | 0.657 | [0.603, 0.711] | 0.765 | [0.714, 0.816] | 0.538 | [0.474, 0.603] | 0.466 | [0.402, 0.531] |
| 18-20 y | 0.883 | [0.829, 0.937] | 0.637 | [0.569, 0.705] | 0.802 | [0.746, 0.859] | 0.478 | [0.402, 0.555] | 0.537 | [0.460, 0.613] |
| 21-23 y | 0.918 | [0.861, 0.975] | 0.734 | [0.665, 0.803] | 0.805 | [0.734, 0.877] | 0.517 | [0.418, 0.616] | 0.496 | [0.396, 0.595] |
| 24-26 y | 0.895 | [0.794, 0.997] | 0.638 | [0.514, 0.762] | 0.782 | [0.671, 0.892] | 0.531 | [0.391, 0.670] | 0.487 | [0.340, 0.633] |

Note. DD: Disease Duration; MO: Mobility; SC: Self-care; UA: Usual Activities; PD: Pain/Discomfort; AD: Anxiety/Depression. The rating 2 and 3 (“some” and “severe” problems) were aggregated and compared with rating 1 (no problem). Only the probability of reported problems is shown in the table

## **Supplementary Fig 1**. Predicted probability of each rating level selection in different EQ-5D subdimensions

| A. Mobility  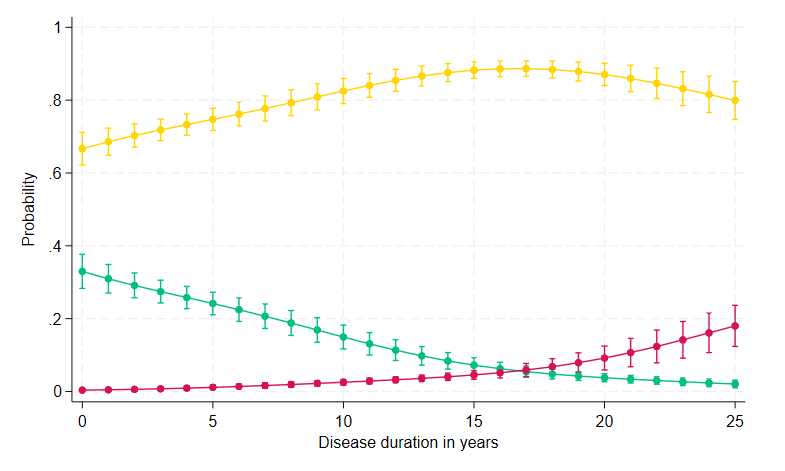 | B. Self-care  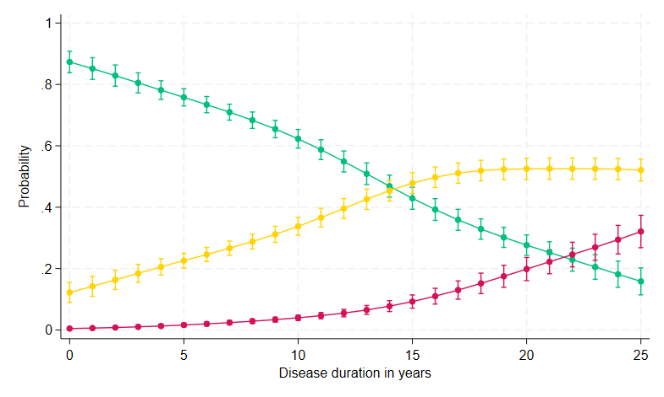 |
| --- | --- |
| C. Usual Activity  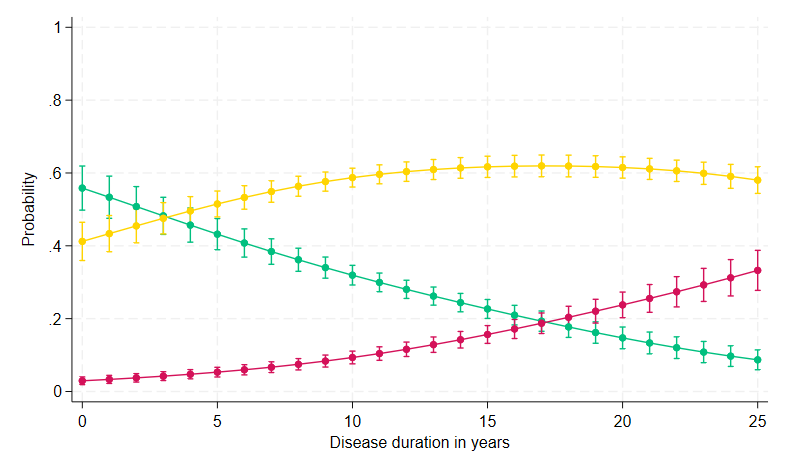 | D. Pain/Discomfort  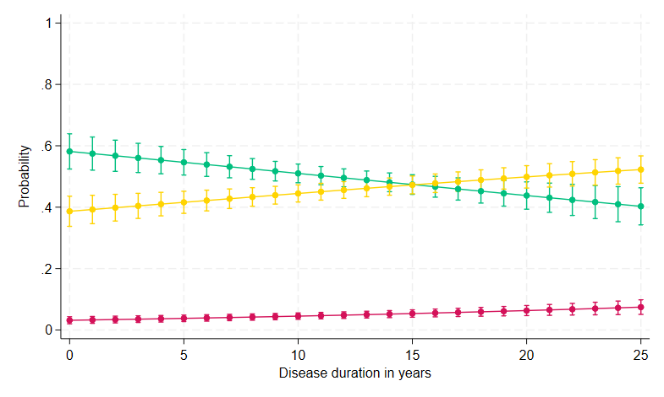 |
| E. Anxiety/Depression  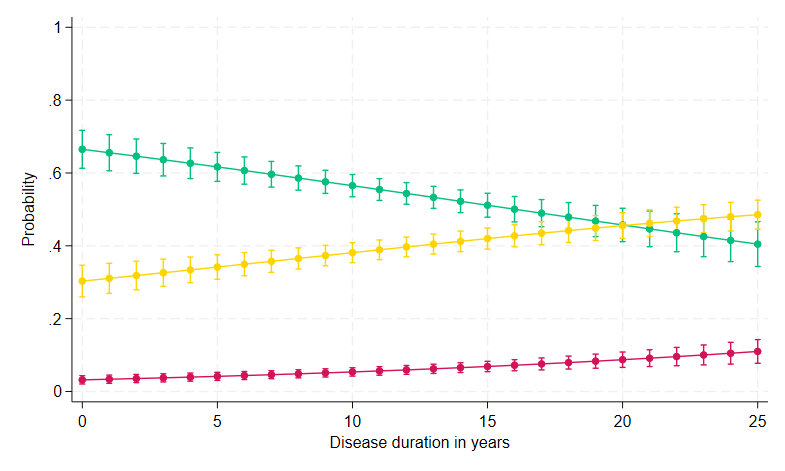 | 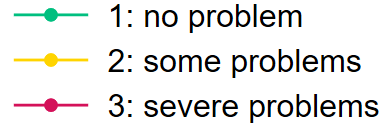 |

Note. The probabilities at each point in the x axis disease duration adds up to 100%.

## **Supplementary Fig 2.** Percentage of each subdimension rating levels throughout the disease duration at baseline


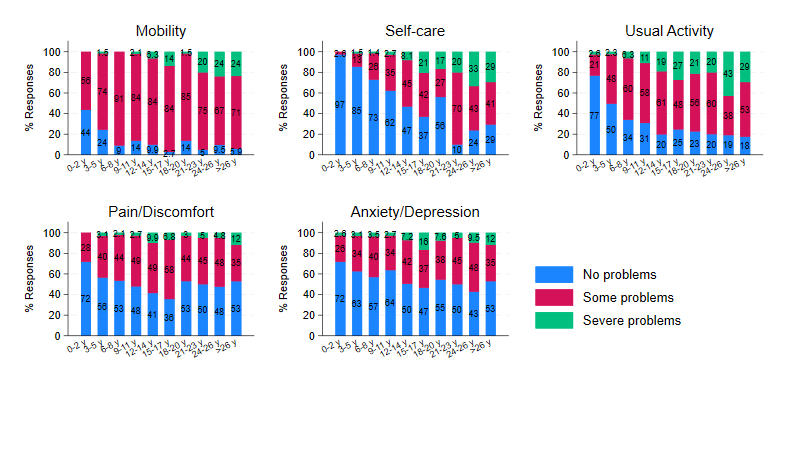


Note. The frequency of each subdimension rating at baseline divided by disease duration. This reflects the average HRQoL ratings of the cohort.

## **Supplementary Fig 3**. Depressive symptom severity levels throughout the disease duration on different visitations


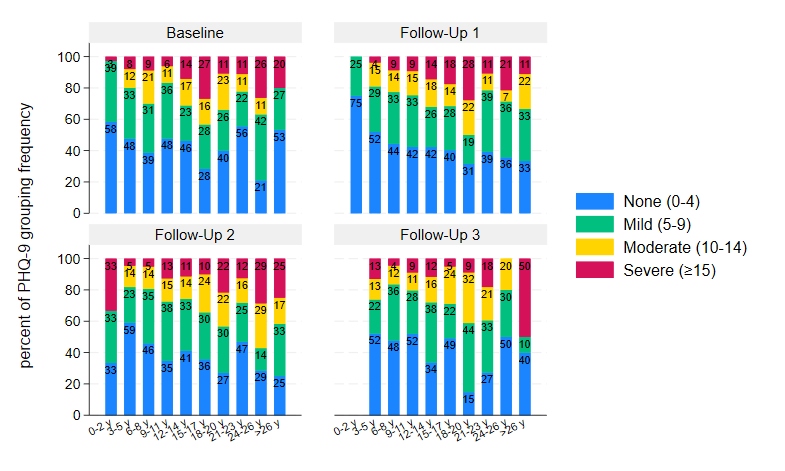


Note. The sample is predominated by patients with none to mild depressive symptoms
