# Supplementary figures and images for: Longitudinal description of health-related quality of life and depressive symptoms in polyQ spinocerebellar ataxia patients
Source: J Neurol. 2025 Apr 9;272(5):323. doi: 10.1007/s00415-025-13024-0 (PMC11982069; doi:10.1007/s00415-025-13024-0)

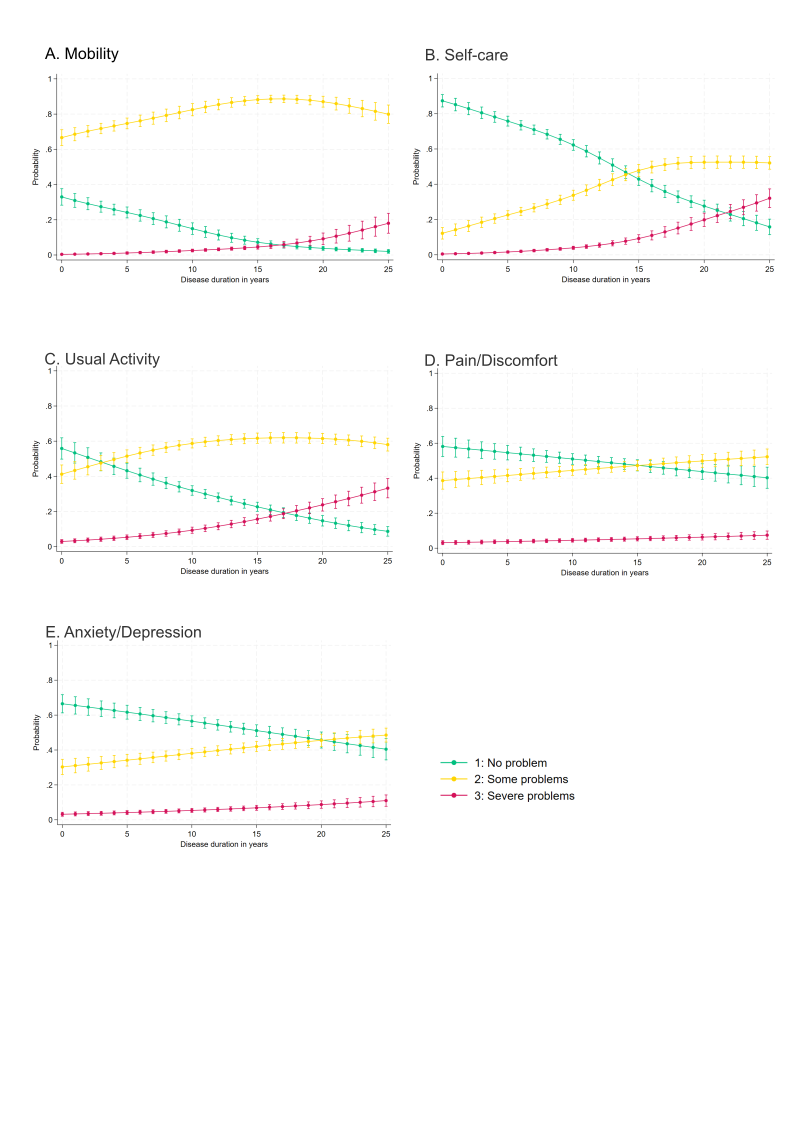

Supplement: Supplementary file 2 — Supplementary file2 (TIFF 3484 KB) [file 415_2025_13024_MOESM2_ESM.tiff]

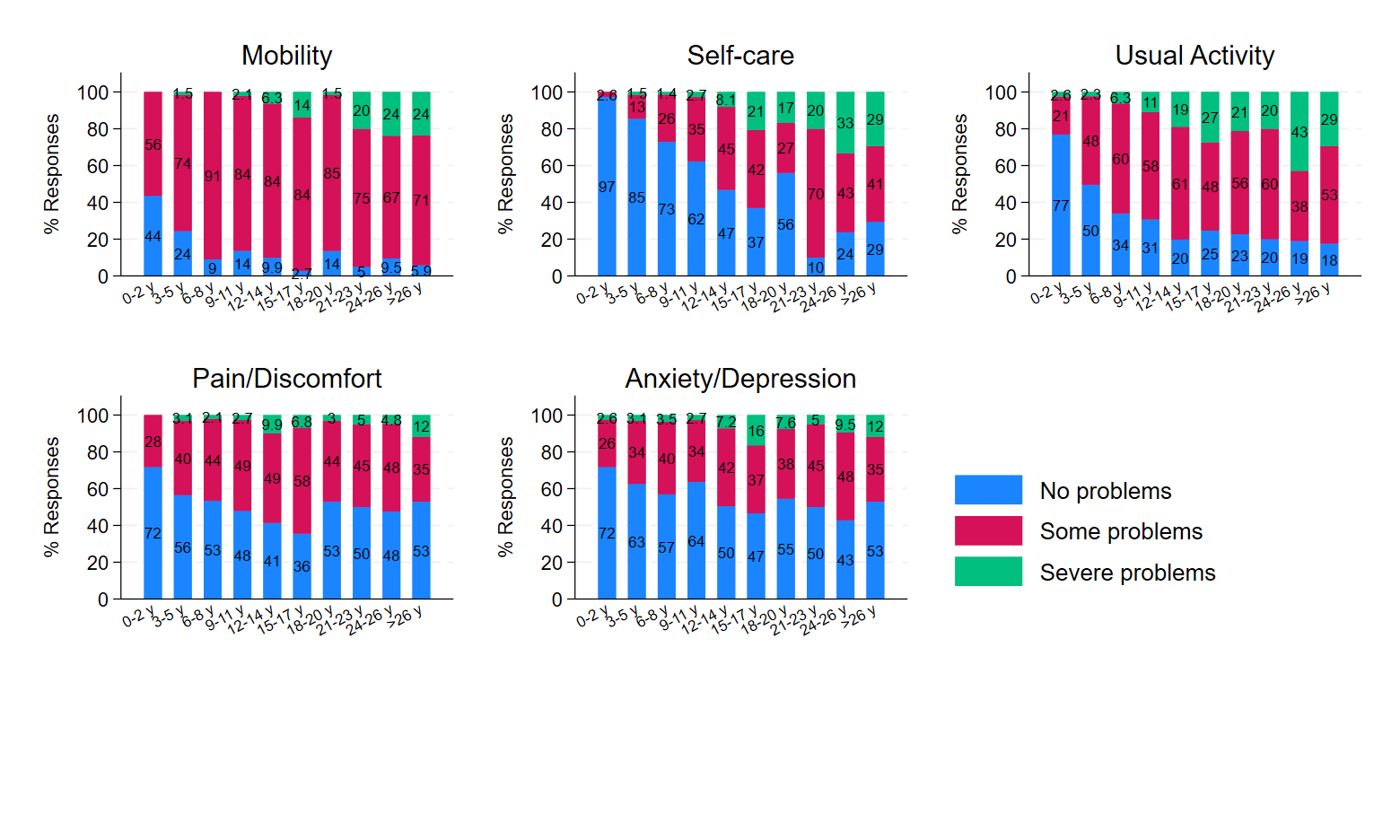

Supplement: Supplementary file 3 — Supplementary file3 (TIFF 4281 KB) [file 415_2025_13024_MOESM3_ESM.tif]

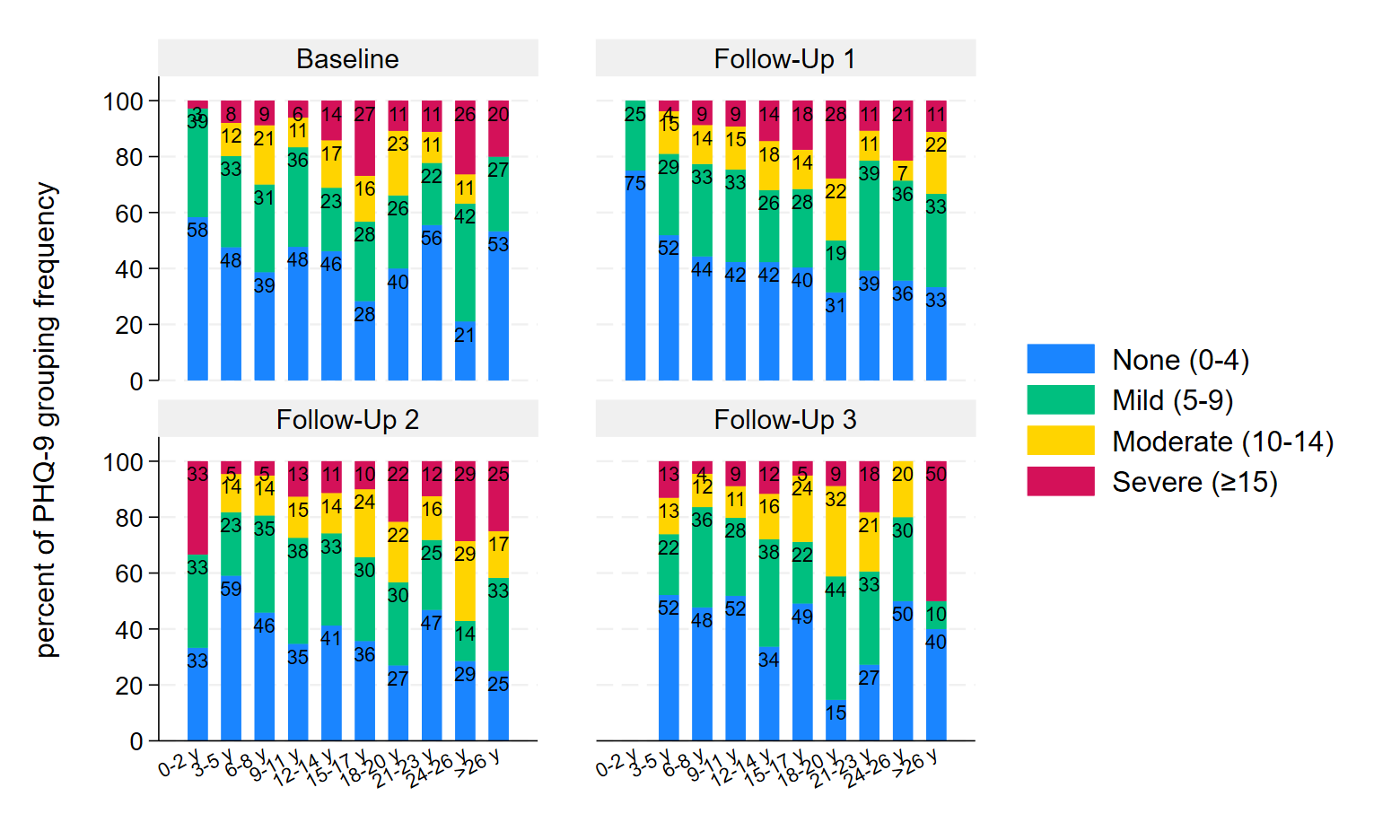

Supplement: Supplementary file 4 — Supplementary file4 (TIFF 4281 KB) [file 415_2025_13024_MOESM4_ESM.tif]
